# Censored at the Nanoscale

**DOI:** 10.3389/fmicb.2016.00253

**Published:** 2016-02-26

**Authors:** Boris L. T. Lau, Caitlyn S. Butler

**Affiliations:** Department of Civil and Environmental Engineering, University of Massachusetts AmherstAmherst, MA, USA

**Keywords:** nanomedicine, nanoparticles, quorumsensing, quorumquenching, acylhomoserine lactone, cyclodextrins, biofilms

As soon as researchers uncovered microorganisms' abilities to communicate, efforts began to control the conversation. Among other cellular functions, quorum-sensing is implicated in biofilm formation, a problematic phenomena in a variety of settings such as persistence of infections (Costerton et al., 1999; Rutherford and Bassler, 2012) and biofouling of water- and wastewater-treatment membranes (Flemming, 1997; Ramesh et al., 2007; Yeon et al., 2008; Shrout and Nerenberg, 2012). Cell-to-cell communication has also been documented in biofilm dispersal leading to further propagation of biofilms within these systems (Solano et al., 2014).

Many efforts to interrupt and quench quorum-sensing have exploited the knowledge of signaling systems using specific model organisms, most notably *Pseudomona aeruginosa, Staphylococcus aureus*, and *Vibrio fischeri* (Stevens and Greenberg, 1997; Miller and Bassler, 2001; Schuster and Greenberg, 2006; Novick and Geisinger, 2008). However, specific approaches have been developed to target and block gene-regulation or to inactivate receptor proteins, however, these approaches may have limited effects in mixed-community biofilms. Non-specific quenching of quorum-sensing molecules may have broader impact. Microbially-generated enzymes such as lactonases and acylases can hydrolyse *N*-acyl-L-homoserine lactones (HSLs), and interfere with communication (Park et al., 2006; Uroz et al., 2008; Romero et al., 2011). Both naturally-derived-, such as rosamaric acid and vanillin (Walker et al., 2004; Choo et al., 2006; Ponnusamy et al., 2009), and synthetic-chemicals, including brominated furanones, have been shown to effectively inhibit biofilm formation. The delivery of effective and non-toxic quorum-sensing inhibitors however, remains a challenge in managing biofilms. In a recent Frontiers in Microbiology article, Miller et al. (2015) introduce a different and unique approach that exploited the slightly-hydrophobic core of a beta-cyclodextrin (β-CD) to non-specifically bind HSLs, and quench the signaling between *V. fischeri* cells. What makes Miller et al.'s approach stand apart is they immobilized the β-CD on the surface of silicon dioxide nanoparticles^1^ (Si-NPs).

Like quorum-inhibition and quenching approaches, NPs, on their own, offer opportunities for biofilm control. NPs are being explored to inhibit or prevent biofilm formation on surfaces (Kalishwaralal et al., 2010; Tran and Webster, 2011) as well as increase biofilm vulnerability to antibiotics (Applerot et al., 2012; Radzig et al., 2013). Miller et al. have demonstrated a proof-of-concept approach, using NPs for quorum-quenching. However, NP penetration into biofilms should be carefully considered. Diffusion is reported as a function of NP size, surface charge, biofilm density and thickness. Self-diffusion of NPs is reported to decrease exponentially with square of the NP radius and negatively-charged NPs is reduced further (Peulen and Wilkinson, 2011).

One of the interesting findings by Miller et al. is that the state of β-CD (i.e., unbound vs. immobilized on 15 or 50 nm Si-NPs) greatly affects its ability to impact QS. It is important to recognize that surface-immobilized organics possess very different properties than unbound ligands. For example, the apparent acid dissociation constant (pKa) of 11-mercaptoundecanoic acid (MUA) lies between ~4.8 (when the free molecules are in solution) to ~10 (when immobilized on a flat surface). When MUA is immobilized on a relatively small NP (surface with a high curvature), a mere change of NP diameter from 4 to 7 nm could result in a change of pKa by as much as one pH unit.(Wang et al., 2011) On a non-spherical NP surface (e.g., nanorod or nano-dumbbell), organic molecules tethered onto regions of different geometric curvature would experience different degrees of confinement, which ultimately translate into location-specific chemical properties (Walker et al., 2013).

NP ligand properties (e.g., size, density, type, and orientation) have been shown to greatly impact drug delivery (Bandyopadhyay et al., 2011; Wang et al., 2014; Amin et al., 2015). Depending on the sizes and shapes of NPs, ligand density could affect *in-vitro* cellular internalization and/or *in-vivo* biodistribution (Reuter et al., 2015). β-CD, being used as a scaffold for ligands, is capable of regulating ligand properties. The primary hydroxyl group located on the narrower ring of β-CD can be selectively modified by various biomolecules (e.g., peptides, ssDNA). For example, the average and localized lysine density on β-CD can be tuned to regulate the adsorption of proteins (Shi et al., 2015).

Distinct control of ligand density is an important design parameter for NPs to be a more effective sponge of QS signaling molecules. An optimal non-saturating ligand density has been found to exist (across different sizes of NPs and targeted receptors) and that identifying this density is crucial for various applications of nanomedicines (Poon et al., 2010; Elias et al., 2013). Increasing the average number of ligands per NP will greatly reduce the inter-ligand spacing. An overcrowding of ligands on NP surface could potentially (1) create a competitive sorption environment for multiple ligands to bind to a single receptor and (2) prevent ligands from obtaining the necessary conformation for binding (Elias et al., 2013).

Overall, future improvements in NP design to facilitate quorum quenching lies in: (1) the careful selection of NPs with appropriate sizes and shapes and (2) the development of novel bioconjugation strategies (e.g., click chemistry) to maintain the functional properties of ligands. These advances should propel NPs into a prominent position in the toolbox for stopping the microbial chatter.

## Author contributions

CB provided an overview of quorum sensing and the different approaches of its interruption. BL discussed some of the important nanoparticle design considerations for effective quorum quenching.

### Conflict of interest statement

The authors declare that the research was conducted in the absence of any commercial or financial relationships that could be construed as a potential conflict of interest.

## Footnotes

^1^Generally speaking, nanomaterials are often defined as a material that consists of particles with one or more dimensions in the size range 1–100 nm. It is important to note that a ‘one size fits all’ definition may fail to capture what is important. The change in reactivity at the nanoscale depends critically on the particular material and the context.
